# Supplementary material for: Improved Peripheral and Hepatic Insulin Sensitivity after Lifestyle Interventions in Type 2 Diabetes Is Associated with Specific Metabolomic and Lipidomic Signatures in Skeletal Muscle and Plasma
Source: Metabolites. 2021 Dec 3;11(12):834. doi: 10.3390/metabo11120834 (PMC8708788; doi:10.3390/metabo11120834)
Supplement: Supplementary file 1 [file metabolites-11-00834-s001.zip › Supplementary Methods .pdf]

Supplementary Materials

Supplementary Methods 1: Metabolomic and lipidomic analyses and data processing

Skeletal muscle samples were extracted with methanol/water (90/10, v/v) for metabolomic analyses and chloroform/methanol (2:1, v/v) for lipidomic analyses, both extraction mixtures are spiked with isotope labelled internal standards. Samples were prepared and analysed in designed batches to reduce the methodological biases that may interfere with the interpretation of the results [1], i.e., samples from the same individual were prepared and analyzed in close connection, but with a randomized internal order. In addition, analytical batches were balanced in terms of treatment group and quality control samples (i.e., pooled from all samples, blanks and injection standards) were added to monitor instrument stability.

Prior to GC-TOF/MS metabolomics analysis, a further two-step derivatization procedure was carried out to increase metabolites volatility (silylated) and reduce number of tautomeric form of each carbohydrate (methoxymated) (A et al. 2005). The derivatized samples (1 µL) were analyzed on an Agilent 6890 gas chromatograph equipped with a 10 m × 0.18 mm i.d. fused silica capillary column with a chemically bonded 0.18-µm DB 5-MS stationary phase (J&W Scientific, Folsom, CA). The injector temperature was 270 °C. The column temperature was held at 70 °C for 2 min, increased by 40 °C min^{-1} to 320 °C, and held there for 1 min. The column effluent was introduced into the ion source of a Pegasus III time-of-flight mass spectrometer, GC-TOF/MS (Leco, St. Joseph, MI, USA). The transfer line and the ion source temperatures were 250 and 200 °C, respectively. Ions were generated by a 70-eV electron beam at an ionization current of 2.0mA. The spectra were recorded in the mass range 50–800 m/z at a rate of 30 spectra s^{-1}.

Prior to LC-TOF/MS analyses, the samples were re-suspended in 10 + 10 µL of methanol and water for metabolomic analysis and re-suspended in chloroform/methanol (2:1, v/v) for lipidomic analysis. Aliquots of muscle extracts (2 µL) were injected onto a Waters Acquity UPLC HSS T3 C_{18} column (2.1 × 50 mm, 1.8 µm, Waters, Milford, MA, USA) in combination with a 2.1 mm x 5 mm, 1.8 µm VanGuard precolumn (Waters Corporation, Milford, MA, USA) held at 40 °C. The chromatographic separations of metabolites were carried out using a gradient solvent system consisted of water with 0.1 % formic acid (A) and acetonitrile/isopropanol (75/25, v/v) with 0.1 % formic acid (B) at a flow rate of

0.5 mL min^{-1}. The gradient started at 0.1 % B increasing linearly to 10 % B in 2 min and increased to 99 % B over 5 min which held for 2 min; the proportion of B was decreased to 0.1 % for 0.3 min and was further decreased to 0 % with the flow-rate increased to 0.8 mL min^{-1} for 0.5 min which held for 0.9 min; the initial conditions were restored in 0.1 min before the next injection.

The chromatographic separations of lipids (aliquots of muscle lipid extracts 1 μL) were carried out using a gradient solvent system consisted of mobile phase A (60:40 acetonitrile:water + 10 mM ammonium formate + 0.1% formic Acid) and mobile phase B (89.1:10.5:0.4 isopropanol:acetonitrile:water + 10 mM ammonium formate + 0.1% formic acid) at a flow rate of 0.5 mL min^{-1}. The gradient started at 15 % B increasing linearly to 30 % B in 1.2 min and increased to 55 % B over 0.3 min which held for 3.5 min; the proportion of B was increased to 72 % in 2 min and increased to 85 % in 2.5 min, and further increased to 100 % in 0.5 min. The wash program started after the sample analysis; the initial conditions were restored in 0.3 min before the next injection.

The detection of separated metabolites and lipids was performed using the Agilent 6550 Q-TOF mass spectrometer equipped with a jet stream electrospray ionization (ESI) source, operating in both positive and negative ion modes. A reference interface was connected for accurate mass measurements and reproducibility. Full scan MS spectra were collected in a centroid mode over the mass range 70-1700 m/z with an acquisition rate of 4 spectra s^{-1}. The capillary voltage was set at +4 kV and –4 kV with nozzle voltages of +300 V and –300V for positive and negative ion modes, respectively. Other MS parameters were applied as follows: gas temperature was set at 150 °C, drying gas flow was 16 L min^{-1}, and the pressure of nebulizer gas was 35 psig. The sheath gas flow was kept at 11 L min^{-1}with a temperature of 350 °C. The voltages of the fragmentor, skimmer, and octopole RF peak voltage were 380 V, 45 V and 750 V, respectively. The LC-TOF/MS data acquisition was performed in global profiling mode including the initial MS/MS scanning for the further targeted and untargeted data analysis. Additional targeted and untargeted MS/MS analyses of quality control samples were performed for targeting metabolites included in the in-house database and for elucidating the structures

of untargeted metabolites, respectively. Data were acquired with MassHunter Acquisition Software B.08.00.

Data Processing

The processing of GC-TOF/MS data and extraction of putative metabolites are described by Chorell et al [2]. Briefly, an in-house MATLAB script was used for the extraction of putative metabolites by matching the mass spectra and retention indices to in-house mass spectral library at the Swedish Metabolomics Centre and the publicly available Max Planck Institute library in Golm. The processing of LC-TOF/MS data and extraction of putative metabolites and lipids were performed by MassHunter Profinder version B.08.00 in combination with Qualitative Analysis software version B.07.00, PCDL manager version B.07.00 and Mass Profiler Professional™ 13.0 (all from Agilent Technologies Inc., Santa Clara, CA, USA). Annotation of putative metabolites were done by matching the retention time (MS and MS-MS spectra) against the in-house metabolite and lipid library. The match tolerance of masses deviation was set to ± 20 ppm and retention time deviation was adjusted to ± 0.15 min, respectively. Features were firstly filtered by manual inspection of the data chromatographic and mass spectral profiles and removal of non-Gaussian peaks as well as peaks with deviating % RSD-values > 10 ppm (Mass) and % RSD > 20 (target score of molecular feature extraction). Next, to remove noisy features and background, we calculated an OPLS model for the extracted features using the concentration of the dilution series of QC samples as response. Features with VIP < 1 and with a negative correlation to the dilution series of QC samples were removed. By using metabolic profiling mode, numerous of unknown putative metabolites and lipids, not available in current databases, could be quantified and included in sample comparison modelling.

References

[1] Jonsson P, Wuolikainen A, Thysell E, et al. (2015) Constrained randomization and multivariate effect projections improve information extraction and biomarker pattern discovery in metabolomics studies involving dependent samples. Metabolomics 11(6): 1667-1678

[2] Chorell E, Ryberg M, Larsson C, et al. (2016) Plasma metabolomic response to postmenopausal weight loss induced by different diets. Metabolomics 12(5). ARTN 85
10.1007/s11306-016-1013-x
